# Data Mining of Microarray Datasets in Translational Neuroscience

**DOI:** 10.3390/brainsci13091318

**Published:** 2023-09-14

**Authors:** Lance M. O’Connor, Blake A. O’Connor, Jialiu Zeng, Chih Hung Lo

**Affiliations:** 1 College of Biological Sciences, University of Minnesota, Minneapolis, MN 55455, USA; ocon0436@umn.edu; 2 School of Pharmacy, University of Wisconsin, Madison, WI 53705, USA; baoconnor2@wisc.edu; 3 Lee Kong Chian School of Medicine, Nanyang Technological University, Singapore 308232, Singapore; jialiu.zeng@ntu.edu.sg

**Keywords:** microarray analysis, biological samples, messenger RNA (mRNA), microRNA (miRNA), circular RNA (circRNA), long non-coding RNA (lncRNA), multi-omics integration, translational neuroscience, biomarker discovery, therapeutic development

## Abstract

Data mining involves the computational analysis of a plethora of publicly available datasets to generate new hypotheses that can be further validated by experiments for the improved understanding of the pathogenesis of neurodegenerative diseases. Although the number of sequencing datasets is on the rise, microarray analysis conducted on diverse biological samples represent a large collection of datasets with multiple web-based programs that enable efficient and convenient data analysis. In this review, we first discuss the selection of biological samples associated with neurological disorders, and the possibility of a combination of datasets, from various types of samples, to conduct an integrated analysis in order to achieve a holistic understanding of the alterations in the examined biological system. We then summarize key approaches and studies that have made use of the data mining of microarray datasets to obtain insights into translational neuroscience applications, including biomarker discovery, therapeutic development, and the elucidation of the pathogenic mechanisms of neurodegenerative diseases. We further discuss the gap to be bridged between microarray and sequencing studies to improve the utilization and combination of different types of datasets, together with experimental validation, for more comprehensive analyses. We conclude by providing future perspectives on integrating multi-omics, to advance precision phenotyping and personalized medicine for neurodegenerative diseases.

## 1. Introduction

Over the past few decades, methods for quantifying the transcriptome have developed and expanded from microarray gene expression and quantitative polymerase chain reaction [1,2] to bulk RNA-seq and single-cell or single-nucleus RNA sequencing (sc/snRNA-seq) [3]. RNA-seq techniques have been at the forefront of studies aimed at understanding the heterogeneity of neurological diseases, including Alzheimer’s disease (AD), Parkinson’s disease (PD), and multiple sclerosis (MS) [3,4]. It also has the unique ability of being able to detect novel sequences and splice variants [3,5]. However, RNA-seq methods are generally more labor intensive in data analysis and not as cost efficient in terms of data storage, and they may possess transcript length bias, which is currently mediated by long-read sequencing [5]. Although microarray gene expression analysis is limited to transcripts that are already established for the model organism being analyzed, it is able to detect highly varied genes [6]. Despite the technical differences, results from microarray and RNA-seq analyses have been shown to be highly consistent with each other [7]. In the context of data mining, microarray analysis is still widely adopted due to its low cost, high efficiency, limited bias [8], greater statistical power [9], and vast number of public neuroscience datasets available for data mining [3,10].

Data mining enables the utilization and comparison of deposited datasets containing high-dimensional features to efficiently acquire information related to translational neuroscience, which can be used to generate new hypotheses and may be validated experimentally [11]. When mining for transcriptomics data, it is important to take into account the type of RNA data and biological samples used in the analysis. For example, to probe for the pathogenic mechanisms of neurodegenerative diseases, the RNA profiles of post-mortem brain tissues from patients will provide insights into the specific alterations of the biological pathways that might play key roles in disease pathogenesis. On the other hand, regarding biomarker discovery, alterations in the RNA signatures from biological samples that can be obtained non-invasively, such as blood, could be used. Changes in the RNA profiles of cerebrospinal fluid (CSF) are sometimes utilized for biomarker discovery, although it is worth noting that CSF extraction can be invasive. Hence, there is a pressing need for the further investigation and establishment of blood biomarkers of neurodegenerative diseases [12,13]. Finally, drug discovery in translational neuroscience requires the testing of therapeutics in physiologically relevant models. Induced pluripotent stem cells (iPSCs) derived from patients are important models that can be used to test how therapeutics alter RNA profiles, protein expression, and cellular functions.

In this review, we will discuss the various biological samples that are commonly used for transcriptomic analysis of neurological diseases, with a specific focus on their limitations and advantages. We then discuss the pipeline for mining of microarray gene expression data, including the identification of datasets, quality control checks, statistical analysis, and functional annotations of genes. We further summarize the various types of RNA samples, including protein coding messenger RNA (mRNA) and non-coding RNA such as microRNA (miRNA), circular RNA (circRNA), and long non-coding RNA (lncRNA), that have been studied using microarray analysis for translational neuroscience applications. Furthermore, we discuss the gaps to be bridged between microarray and RNA-seq techniques and highlight how these two methods are complementary to each other. We conclude by providing future perspectives for the advancement of multi-omics integration, precision phenotyping, and personalized medicine for neurodegenerative diseases.

## 2. Biological Samples for Microarray Analysis

The selection of biological samples for data mining forms the core basis of both bioinformatics and experimental analyses, and it determines the outcomes and conclusions of studies. In neuroscience, these biological samples mostly consist of brain tissues, CSF, peripheral blood, as well as human stem cells. Samples collected from healthy controls or patients with neurodegenerative diseases are subjected to an array of quantitative and qualitative biological measurements, such as microarray characterizations and image analysis. The results of these measurements are interpreted to understand the brain functionality throughout various stages of disease [14,15]. It is important to classify biological samples based on basic demographic information, as well as genotypes (e.g., patients containing pathogenic mutations), phenotypes (e.g., observable characteristics that arise from the diseases), and clinical outcomes (e.g., patient-derived characteristics such as the Braak stage) of the subjects [16]. Another consideration lies in the ease of obtaining the biological samples for analysis, including the experimental procedures involved and whether it is invasive. Furthermore, the selection of biological samples should also be determined based on the applications of the studies, whether it is for biomarker identification, drug discovery, or the elucidation of disease mechanisms.

### 2.1. Brain Tissues

The use of human post-mortem brain tissues provides direct observations concerning the pathology and disease state when the patient is deceased. However, the inability to obtain brain tissue samples from living patients over time, and only during the last stage of life, creates bias and cannot be used to assess the initial stages of the disease. It is also difficult to elucidate the course of disease progression that determines clinical outcomes and disease phenotypes associated with the patients [17,18]. Furthermore, the main issues with using tissue samples for analysis lie in tissue heterogeneity, including diversity and variability, as well as low reproducibility across patient subjects [19,20]. Heterogeneity associated with neurodegeneration is further supported with a machine-learning technique that analyzes imaging datasets to reveal data-driven disease phenotypes, temporal progression, and trajectories that are distinct across patients [21]. Due to these abnormalities, a combined analysis of multiple datasets, containing different batches of samples and large number of patients, would provide a more accurate analysis. The increased utilization of data mining of brain tissue-associated microarray datasets holds promise for biomarker discovery and enhanced understanding of disease mechanisms in neurological disorders, leading to improved prognosis [22,23].

### 2.2. CSF and Peripheral Blood

To profile living patients, CSF and peripheral blood are often used as biological samples due to their extractability and diagnostic applications [24,25], and they are less likely to be affected by heterogeneity [19]. Unlike peripheral blood with expansive applications, the primary application of CSF is for the detection and diagnosis of neurological diseases [26]. Among several other candidates, established CSF biomarkers such as β-amyloid and tau can be used for the early diagnosis of AD [27], whereas α-synuclein and neurofilament light chains have been shown to aid the diagnosis of PD and MS, respectively [28,29,30,31]. On the other hand, establishing blood biomarkers has been a highly sought after strategy due to their extremely low invasiveness, low cost, and accessibility [32,33,34]. Currently, work is being conducted in order to increase the precision of measurements and to increase the corroboration between blood biomarkers and established CSF biomarkers [35]. This suggests that blood biomarkers may be used in clinical practice for diagnosing neurodegenerative diseases in the near future. However, for blood biomarkers to be fully implemented and consistent with observations from other biological samples, such as brain tissues, more correlation studies need to be conducted and new analysis methods need to be developed to take into account the variability between samples and individual patients [35,36]. Multiple studies utilizing peripheral blood have been focusing on examining miRNA expression levels due to their biomarker-quality characteristics [37,38,39]. More specifically, miRNAs are small non-coding RNAs that are being utilized for analyzing dysregulated genes due to their abundance, tissue specificity, and stability [40]. Currently, CSF and peripheral blood are often used in combination for disease evaluation, with the disease state being confirmed by established CSF biomarkers, and differentially expressed genes (DEGs) are isolated from peripheral blood to help reinforce the credibility of the proposed blood biomarkers [26,41]. With the need to provide treatment for asymptomatic patients with neurodegenerative diseases, such as AD, as well as to screen for risk in large numbers of young individuals, the development of biomarkers is shifting from a focus on CSF to peripheral blood, due to the ease of extractability and decreased invasiveness [42].

### 2.3. Human Stem Cells

The use of human stem cells is on the rise due to the high applicability of these cells in understanding disease mechanisms, as well as in regenerative therapy [43]. Stem cell therapy offers the ability to regenerate neural tissue and ameliorate the effects of neurodegeneration [44,45]. In addition, human iPSCs from patient fibroblasts can be differentiated to derive a vast source of central nervous system (CNS) cell types and contribute to a generation of multicellular organoids [46]. Other advantages associated with using stem cells include their ability to proliferate while maintaining developmental potential, the ease of modifying their genes, and the direct modeling of human biology without species-specific confounding factors [47]. Compendium-based big data approaches have been proposed to characterize the identity of each differentiated cell type from stem cells, which holds the key to understand the molecular events associated with the cell type and its biological applications [48,49,50]. For example, it has been shown that Aβ secreted from early-onset familial AD iPSC-derived neurons, was highly responsive to γ-secretase inhibitors and modulators, indicating their potential use for the identification and validation of candidate drugs [51]. The use of stem cells in model systems has also led to an increase in the utilization of iPSCs for drug screening and in vitro drug analysis [52]. These studies illustrate the potential of using datasets obtained from human stem cells for bioinformatic analysis to provide extensive insights into biomarker discovery and therapeutic development. Lastly, stem cells play a key role in regenerative medicine, and it is important to understand the mechanisms that regulate regeneration across different species and in different tissues. Recently, a Regeneration Roadmap database has been constructed which contains a comprehensive and systematic collection of gene expression and omics data associated with regenerative biology, and it can facilitate data mining studies [53].

In addition to the abovementioned biological samples, bioinformatic analysis also utilizes other samples including plasma, urine, feces, gut microbiome, mucus, saliva, and sputum to study metabolic changes of metabolites [54,55,56,57,58,59]. One study has shown that gut microbiome samples can be isolated and quantified using sequencing to study MS [60]. Additionally, gut microbiome alterations have been shown to modulate CNS autoimmunity in animal studies [60,61]. In vitro cell lines, as well as in vivo models, including transgenic and knock-in mice, have also been used to validate human data acquired from mining datasets associated with neurodegenerative diseases [62]. To this end, the array of biological samples that can be used for bioinformatics analysis is vast. A combination of datasets from various types of samples could be utilized for integrated analysis to understand the biological changes in localized regions (e.g., brain tissues) or in circulation (e.g., CSF and/or blood). It may also be used to understand the correlation and association within physiological systems in response to treatments, drug responses, and disease progression. Therefore, it is important to pinpoint the research question of interest to ensure the usage of appropriate datasets for computational analysis, and so that they fit into the correct biological context for meaningful interpretation. In many instances, the utilization of multiple datasets of various biological samples, in conjunction with experimental validation, is necessary to confirm findings.

## 3. RNA Based Microarray Gene Expression Analysis

**Figure 1:**
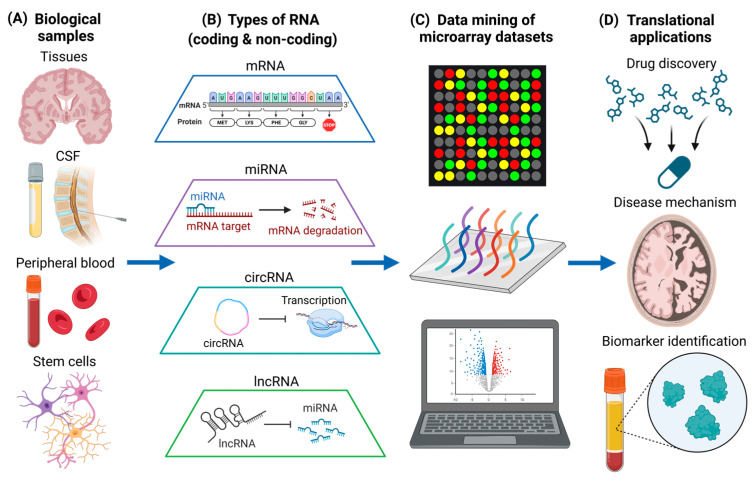
Data mining of different types of RNA in various biological samples for translational neuroscience applications. (**A**) Various types of biological samples, including post-mortem brain tissues, CSF, peripheral blood, and human stem cells. (**B**) Different types of coding (mRNA) and non-coding RNA (miRNA, circRNA, and lncRNA) obtained from biological samples. (**C**) Data mining of microarray datasets associated with neurodegenerative diseases. Different genes detected by the microarray analysis are illustrated by different colors. (**D**) Translational neuroscience applications including drug discovery, the elucidation of disease mechanisms, and biomarker identification. The figure was created using BioRender.

AD and PD are the most common neurodegenerative diseases in the world, and they are characterized by progressive neuron loss [63,64]. On the other hand, MS is a prevalent neuroinflammatory and neuroimmunological disorder characterized by the loss of myelination in the CNS. It also has a neurodegenerative component in the progressive phase which currently does not have effective treatments [65]. Pathogenic mechanisms of neuroinflammation and neurodegeneration includes the dysregulation of biological processes, such as altered signaling pathways [66,67,68], as well as mutant protein production and toxic protein aggregation [69,70,71]. While targeting aberrant signaling pathways and toxic protein aggregates represent important therapeutic strategies [72,73,74], gene-level interventions can also be useful for treatment of neurodegenerative diseases. This is especially when preventing the expression of toxic gain-of-function genes does not detrimentally affect homeostatic cellular processes [75]. Therefore, it is vitally important to understand the changes in RNA expression of different biological samples in diseased states (Figure 1A), and to understand how alterations in RNA levels could have potential therapeutic efficacy. In this section, we will discuss the different types of RNA that are quantified using microarray (Figure 1B) and current microarray-based data mining studies that are associated with neurodegenerative diseases (Figure 1C), which can provide more insights into translational neuroscience applications (Figure 1D).

### 3.1. Pipeline for the Data Mining of Microarray Datasets

**Figure 2:**
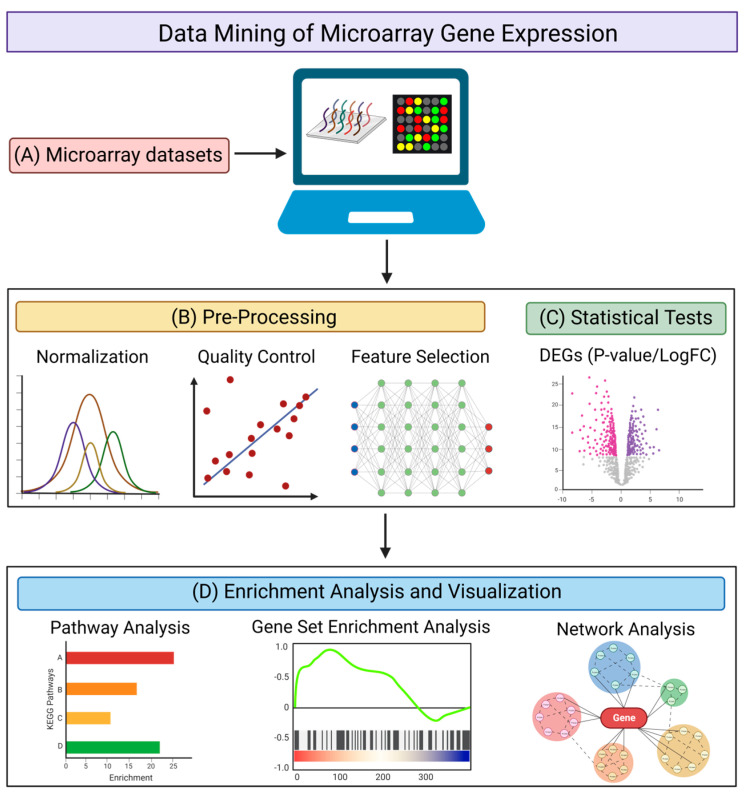
Pipeline for the data mining of microarray gene expression. (**A**) Searching for suitable microarray datasets to be analyzed using web-based tools or command line scripts. Different genes identified by microarray analysis are represented by different colors. (**B**) Pre-processing of datasets via normalization, quality control, and feature selection. (**C**) Statistical tests to obtain DEGs with corresponding P-values (significance) and LogFC (fold-change). Downregulated genes are illustrated in red and upregulated genes are illustrated in purple. (**D**) Enrichment analysis and visualization using pathway analysis, gene set enrichment analysis (GSEA), and network analysis to provide a biological interpretation of the DEGs. Different pathways or functional annotations of the DEGs are illustrated by different colors. The figure was created using BioRender.

The microarray method has been one of the most commonly used methods of transcriptomic analysis. It is used for identifying protein-encoding transcripts or non-coding RNAs that are differentially expressed in diseased states (as compared with healthy controls) by quantifying various RNA expression levels [76]. There are multiple databases archiving microarray datasets [77,78,79,80], with the Gene Expression Omnibus (GEO) database being the predominant repository [79]. The GEO database has a built-in tool, GEO2R, which is a graphical user interface that can be used to compare two or more groups of samples to identify the DEGs with statistical significance [79]. When isolating DEGs from the data mining of transcriptomic datasets, using a web-based tool such as GEO2R, or a command line-based analysis method using R scripts (Figure 2A), it is important to take into account the necessary quality control steps (Figure 2B) and statistical methods (Figure 2C) for the analysis. For quality control, it may be necessary to normalize the raw data [81], to perform a quality control assessment of the alignment, and to account for any contaminating species [82]. Feature selection may be required to remove genes that serve no biological purpose due to consistent expression across all samples [83,84], depending on the distribution of expression values of the samples used. For statistical analysis, using GEO2R as an example, it utilizes the R studio limma package for a differential analysis, where empirical Bayes moderated *t*-statistics and associated *P*-values, together with fold change values, are produced and used to evaluate the significance and extent of gene expression changes between diseased samples and healthy controls [85]. GEO2R also provides several graph plotting functions, such as the production of volcano and box plots, as well as uniform manifold approximation and projection (UMAP), that provides a further understanding of the expression level of genes and their expression changes under diseased conditions. Venn diagram analysis can also be performed to obtain overlapping DEGs across different datasets concerning similar disease conditions, in order to increase the stringency when determining significant genes with expression changes.

After obtaining the DEGs, there are multiple web-based and application-based programs that are available to elucidate the functional annotations of the DEGs and to provide insight into their role in disease mechanisms (Figure 2D). Tools aimed at conducting pathways analysis and identifying functionally enriched biological processes include the Database for Annotation, Visualization, and Integrated Discovery (DAVID) [86], Ingenuity Pathway Analysis (IPA) [87], Gene Set Enrichment Analysis (GSEA) [88], Centrality-based Pathway enrichment (CePa) [89], Signaling Pathway Impact Analysis (SPIA) [90], FunRich [91], and ExpressAnalyst [92]. Such tools are used to identify specific genes that are involved in biological processes that may pertain to disease pathogenesis, and to investigate how those biological processes are dysregulated under disease conditions compared with control conditions. Although the isolated DEGs are the inputs for most analysis tools, it is important to note that GSEA not only takes into account the DEGs and their expression values, but all transcriptomic expression values from each sample, for every gene in the raw data. Using an entire gene set, as opposed to specifically isolated DEGs, enables a more holistic analysis of the dysregulation of genes in diseased states. Programs and tools aimed at providing information on network visualization and specific protein–protein interactions include STRING [93], Cytoscape [94], and NetworkAnalyst [95]. These programs not only enable the isolation of nodes involved in functionally enriched biological pathways, but they also allow the identification of hub genes and assess the extent to which interactions and connectivity occur.

### 3.2. Microarray Analysis of Coding RNA (mRNA)

Microarray analysis has been used to assemble data that are representative of the mRNA expression levels of tens of thousands of genes in different neurodegenerative diseases. It can also identify DEGs in these disease states, which can be further explored to establish therapeutics or biomarkers. Here, we summarize results from data mining studies to elucidate the changes in gene expression in various sample types under different neurological conditions (Table 1).

In AD, a vast number of data mining studies have been conducted to examine altered gene expression in brain tissues (upregulated: *HDAC1* [96], *WWTR1* [97], *ITGB1* [98], *PDGFRB* [99], *PLOD1* [99], *MAP4K4* [99], *NFKBIA* [99,100]; downregulated: *SIRT3* [101], *BDNF* [97], *RAB7A* [98]) and in peripheral blood (upregulated: *VCAM1* [102], *TYK2* [103], *TCIRG1* [103], *PPP3CB* [103], *SNCB* [103], *SACS* [103]; downregulated: *CTSD* [102], *RPL11* [104], *SNCA* [103], *FKBP1B* [103]). Although there is a limited number of studies analyzing CSF samples in microarray analysis, a meta-analysis has reported that *NRGN* is upregulated in the CSF samples of AD patients [105]. Another data mining study utilizing the GEO datasets of human AD brain tissues found that *TYROBP* is a key regulator of pathogen phagocytosis in microglia, and it is upregulated in late-onset AD [106]. Importantly, they further validated their results using a mouse model expressing *TYROBP* in microglia, and they revealed gene expression changes that significantly overlapped with the *TYROBP* network in the human brain [106]. In terms of biomarker discovery, a data mining study found five potential biomarker genes of AD. More specifically, *GSN*, *BDNF*, *TIMP1*, *VLDLR*, and *APLP2* were validated both in bioinformatic analysis using AD GEO datasets of human brain tissues, and in an experimental validation using peripheral blood from AD patients [107].

In PD data mining studies, *LILRB3* and *CSF3R* have been shown to be upregulated [108], and *ICAM1* was shown to be downregulated in whole blood analysis [109]. Additionally, *MAPK8*, *CDC42*, *NDUFS1*, *COX4I1*, and *SDHC* have been shown to be significantly downregulated in PD brain tissues [110]. Another study revealed a significant upregulation of the RNA splicing factor, serine/arginine repetitive matrix 2 (*SRRM2*), in PD through the computational analysis of GEO datasets containing human brain tissues, cells, and whole blood. This was validated experimentally, and the analysis showed a significant upregulation of the upstream exons of *SRRM2* [111]. In MS, a data mining study analyzing CSF found *NLRP3*, *LILRB2*, *C1QB*, *CD86*, *C1QA*, *CSF1R*, *IL1B*, and *TLR2* to be downregulated in MS [112], many of which have been supported and validated experimentally in other studies [113,114,115]. Another study found that similar genes, involved in inflammation or immune responses, existed in MS and COVID-19 patients [116]. Additionally, through network analysis, they found that genes *IL1B*, *P2RX7*, *IFNB1*, *TNF*, and *CASP1* enhanced the network connectivity between the combined gene sets of MS and COVID-19, which is associated with NOD-like receptor signaling [116].

Multiple neurodegenerative studies have been utilizing both microarray and RNA-seq analyses simultaneously, and they have isolated DEGs common to both methods of expression quantification [117,118,119]. For example, a systemic biological approach has also been adopted to integrate RNA-seq datasets for COVID-19 and microarray datasets for AD. This was conducted in order to examine and identify the common transcriptional alterations between COVID-19 and AD patients [119]. This study identified 26 hub genes that could be potential biomarkers and therapeutic targets for COVID-19 patients with AD comorbidities. Another PD study made use of both microarray and RNA-seq datasets to identify 12 significant genes that are commonly dysregulated between the blood and brain, including *C10orf32*, *CCDC82*, *COL5A2*, *COQ7*, *GPNMB*, *HSD17B1*, *KANSL1*, *NCKIPSD*, *PM20D1*, *SP1*, *FRRS1L*, and *IL1R2* [117]. This study demonstrates that both disease processes and systemic disease factors may affect brain and blood cells in a similar manner. The correlation and corroboration between blood and brain transcriptomic data are further exemplified in a recent study in PD [120]. Together, these findings identify molecular signatures in PD patients’ brain and blood for potential pathophysiologic and prognostic importance, and these findings may potentially be applicable to other diseases, including AD and MS.

### 3.3. Microarray Analysis of Non-Coding RNA (miRNA, circRNA, and lncRNA)

In addition to studying mRNA, several non-coding RNAs, including miRNA, circRNA, and lncRNA, are isolated from peripheral blood for diagnosis or mechanistic analysis [121]. With non-coding RNAs making up most of the human genome, they are becoming increasingly sought after in neurodegenerative studies due to their role in neural cell specification during development, and in higher cognitive processes such as memory and plasticity [122]. Similar to mRNA, non-coding RNAs can also exhibit cell type specific expression levels to shape the cellular expression landscape and reinforce cellular identity. Importantly, non-coding RNAs are capable of modulating gene expression at the post-transcriptional level, binding to protein factors, controlling epigenetic mechanisms, and playing key roles in regulating many biological processes [123,124].

For example, miRNA plays a key role in the post-transcriptional gene regulation of mRNA expression [125]. Tissue specific miRNAs are becoming increasingly pursued for biomarker discovery due to their non-invasive extraction, accuracy, reproducibility, and predictability [126,127]. A data mining study that made use of blood datasets from MS patients found that the upregulation of miRNA hsa-miR-328-3p [128], hsa-miR-20a-5p [128], and miR-196 [129] occurred, as did the downregulation of miR-9 [129]. In AD, the dysregulation of miRNA has been identified in peripheral blood (upregulated: hsa-miR-186-5p [130]; downregulated: hsa-miR-125a-3p, hsa-miR-22-3p, hsa-miR-24-3p, hsa-miR-6131, and hsa-miR-125b-1-3p [131]) and in brain tissues (downregulated: hsa-miR-29c [132] and hsa-miR-26b-5p [133]). Another study found that the downregulation of miR-425 was implicated in AD pathogenesis, and a miR-425 deficient transgenic mouse was used to validate the results [134]. The loss of miR-425 in mice induced neuroinflammation and neuronal loss, it exacerbated cognitive impairment, and increased amyloid precursor protein amyloidogenic processing [134]. Interestingly, another study combined GEO datasets from AD tissues and experimental validation using blood samples from AD patients, and they found differential results with regard to hsa-miR-185-5p being upregulated in AD brain tissues, while being downregulated in AD blood samples [135].

With an integrative analysis using both microarray and RNA-seq datasets, seven miRNAs that interact with the eight DEGs were identified in early and late mild cognitive impairment patients [136]. Another study that focused on PD identified changes in miRNA expression in the PD patient’s blood leukocytes when compared with control patients. These changes were identified using RNA-seq techniques, microarray analysis, as well as data mining of GEO microarray data [137]. This study found 16 miRNAs that were differentially expressed in PD patients, and a specific interest in transcription factor *FOXP1*, which they found was implicated in a miRNA-mediated feedback loop that controlled the survival of midbrain dopaminergic neurons. In addition to these findings, it is worth noting the significance of miRNAs and other RNAs in other molecular mechanisms associated with brain diseases, such as vascular dysfunction [138] and retinopathies [139,140], which can deepen our understanding of disease mechanisms, and it may potentially serve as prognostic indicators for neurodegenerative diseases.

In addition to miRNA, circRNA has been suggested to have multiple functions, such as regulating transcription in the nucleus, binding to protein factors, acting as a miRNA “sponge” to compete for miRNA pairing with other RNAs, and being more stable in tissues compared with linear RNAs [141]. Furthermore, circRNA expression is enriched in the brain, aiding in its likelihood to be used in studies associated with neurodegenerative diseases [141]. A bioinformatics analysis using GEO datasets has found circRNAs originating from the following AD pathology-linked genes, *DOCK1*, *NTRK2*, *DLG1*, *KIF1B*, *TRAPPC9*, and *APC*, which are altered in AD [142]. A different study focusing on PD found that miRNA-7 (miR-7), which is bound to by circular the RNA sponge for miR-7 (ciRS-7) [143], is mostly expressed in neurons. Moreover, it represses α-synuclein protein, which ultimately protects against oxidative stress [143]. Although there are limited studies on role of circRNA in MS, an amyotrophic lateral sclerosis (ALS) study performing microarray analysis on the peripheral blood of ALS patients found that circRNAs hsa_circ_0000567 and hsa_circ_0023919 were downregulated, and hsa_circ_0063411 and hsa_circ_0088036 were upregulated [144]. These genes are involved in muscle differentiation in mice, clathrin-mediated endocytosis at neuromuscular junctions, Ago-mediated gene silencing, and there is speculation that they are involved in immune responses.

In addition, lncRNAs are non-coding RNA molecules that are more than 200 nucleotides in length [145], and their function is essential for many biological processes, including epigenetic regulation, cell signal transduction, immune response, and cell proliferation and differentiation. Moreover, their abnormal expression can result in a variety of neurodegenerative diseases [146]. By analyzing GEO datasets, a study has found that lncRNA-XIST was downregulated in the whole blood of PD patients [147]. Many neurodegenerative studies have focused on the specific upregulation of the nuclear paraspeckle assembly transcript 1 (*NEAT1*) under diseased conditions. An AD study found that the lncRNAs LOC100507557 (downregulated), LOC101929787 (upregulated), *NEAT1* (upregulated), and *JAZF1-AS1* (downregulated) were differentially expressed, and they were found to be dysregulated in five distinct anatomical regions of the brain [148]. With the 15-fold upregulation of *NEAT1* in the entorhinal cortex, it is the highest upregulated lncRNA in all the analyzed brain regions and has the potential to serve as a biomarker of AD. Using an integrative analysis consisting of microarray, RNA-seq, and genome-wide association study (GWAS) datasets, a study identified five key lncRNAs associated with AD risk, and they were involved in the regulation of the immune system [149].

### 3.4. Bridging Gaps between Microarray and RNA-Seq Analysis

The experimental aspects of microarray and RNA-seq are similar in terms of how RNA is converted into cDNA. It is followed by signal quantification, although the technical details may provide slightly different information. In microarray analysis, cDNA is fluorescently labelled and hybridized with a complementary strand of a known gene, and the fluorescence release directly corresponds with the level of genetic expression of the specific gene in the biological sample [150]. In sequencing studies, gene expression levels are quantified by counts in RNA-seq, which is equivalent to the number of reads mapped on each gene. It is worth noting that newer forms of RNA-seq can directly sequence individual RNA strands with a method known as nanopore direct RNA-seq (DRS). Nanopore DRS allows for the sequencing of single RNA strands, including nucleotide modifications (e.g., methylation, 5′ end capping, 3′ polyadenylation) and all exons, and it has been used to sequence both coding and non-coding RNAs [151]. In sc/snRNA-seq, unique molecular identifiers (UMIs) are further acquired to provide cell-type specific information of the gene expression [152]. The counts may be varied depending on the covariates of the gene or samples such as library size and gene length. As discussed earlier, RNA-seq can detect splice variants and novel sequences, whereas RNA microarray is limited to established transcripts for the model organism being analyzed, although this difference may not affect studies that do not require this detailed level of information. On the other hand, sc/snRNA-seq techniques are of great interest because they not only provide the average expression level for an ensemble of cells, such as in the typical microarray and RNA-seq analyses [153], but also the ability to quantify gene expression levels in specific cell types [153,154,155,156]. Although single cell microarray analyses have previously been reported [152,157], the resolution and the heterogeneity that can be resolved might not be comparable to current sc/snRNA-seq, and they are dependent on the samples that can be obtained.

In addition to the experimental aspects, there have been major gaps in standardizing data analysis pipelines to process different raw data obtained by the different methods of RNA profiling. Although the data analysis for microarray datasets is more straightforward, as it is directly quantified at the expression level, analyses for RNA-seq and sc/snRNA-seq are more complex, with less standardized protocols, a need for more data storage, and a knowledge of coding [158]. With the increasing availability of publicly accessible transcriptomic datasets, many web-based and application-based tools are being created to aid in the analysis of such high-content data. The GEO2R [159] and Bioinformatics Array Research Tool (BART) [160] are web-based programs that are capable of carrying out statistical DEG analysis on deposited GEO microarray datasets. Web-based tools have also emerged to facilitate RNA-seq analysis such as BEAVR [161], RNAlysis [162], RNAdetector [163], OneStopRNAseq [164], and Integrative Differential Expression Analysis for Multiple EXperiments (IDEAMEX) [165]. They consist of graphical user interfaces to assist in conducting DEG statistical analyses, and to assist with the visualization of the results with RNA-seq data. Furthermore, recent studies have also provided simplified and practical guides [166,167], as well as streamlined scRNA-seq data analysis, such as ScAmpi [168]. Additionally, there are tools that enable the analysis and visualization of sc/snRNA-seq data, including Automated Single-cell Analysis Pipeline (ASAP) [169], and the CanceR Single Cell ExpressioN Toolkit (CReSCENT) [170]. We have included a summary table of the data mining tools and programs used to analyze microarray, RNA-seq, and sc/snRNA-seq datasets (Table 2).

An approach to compare and reconcile microarray and RNA-seq analysis methods, specifically in terms of data mining, is check for the similarities between the specific DEGs identified, or to conduct enrichment analysis of all DEGs to see if there are similar pathways and networks obtained from the respective methods. To quantify the similarities between the data obtained from microarray and RNA-seq methods, studies have been carried out to examine the correlation between the expression intensity values. One study specifically used microarray and RNA-seq analysis to quantify the mRNA expression in human brains, which was collected from the Allen Human Brain Atlas. They found consistent, reproducible measurements between the two methods, with a high correlation between expression values (R = 0.78) [171]. They also showed that RNA-seq scaling factors can be applied to improve the sensitivity of microarrays to detect DEGs. Another study examining the lncRNA expression levels in iPSC-derived neurons illustrated a high correlation (R = 0.64) between the expression values of microarray and RNA-seq analysis [172]. These studies suggest that both are suitable methods used for high-throughput gene expression analysis. It is important to note that there is a possibility for false positives, particularly when using a small sample size [173,174]. Hence, the use of multiple datasets, rigorous analysis methods, and stringent statistical parameters is critical to reduce false positives.

### 3.5. Experimental Validation to Advance Therapeutic Development and Biomarker Identification

The current emergence of the big data era and the outburst of transcriptomic datasets from studies with different experimental conditions, biological samples, disease models, developmental states, and responses to treatments, indicate the need for an accurate and reliable data mining process. This presents the need for a thorough interpretation of the outcomes from data mining and their relevance to true biological observations. To test the effect of the DEGs obtained from data mining, we can either knockout or overexpress the key protein of interest, or we can administer modulators of certain signaling pathways to observe how the cellular systems respond to these alterations. It is also important to check whether any of these treatments are toxic to the cells. Typically, when alterations in protein expression or function correlate with disease progression, it indicates that the protein plays a major role in the disease mechanism. High-throughput screening of small molecules or antisense oligonucleotides that can modulate protein function would lead to a therapeutic discovery. Generally, biomarkers are established based on certain key proteins that can be detected in CSF or blood to provide a prognosis of the disease pathogenesis.

Studies have been conducted to quantify the variation and discrepancies between microarray and RNA-seq data [150,174,175], and programs have been created for their integration [176,177]. With evidence showing the corroboration of results between two methods of data collection [7,150], data mining of microarray datasets remain useful for generating novel hypotheses, validating existing RNA-seq data, or integrating novel RNA-seq analysis. Transcriptomics datasets can be extremely high dimensional and may contain tens of thousands of genes, whereas experimental datasets may only contain tens of genes [178]. Furthermore, different analysis criteria adopted in data mining, such as the biological samples used, the number and combination of datasets analyzed, and the cut-off parameters selected, may cause inconsistent results between studies. Hence, there is a definite need to experimentally validate the results obtained from data mining to ensure their accuracy and usefulness.

## 4. Summary and Future Perspectives

A vast number of studies related to neurodegenerative diseases have made use of microarray datasets for the data mining of transcriptomics data. This is mainly due to the expansiveness and diversity of deposited microarray datasets, as well as the ease of processing due to many accessible web-based analysis tools and established computational pipelines. Additionally, it is important to note the ability of microarray datasets to quantify the expression of non-coding RNA species, including miRNA, circRNA, and lncRNA. With the advancements in RNA sequencing technologies, microarray analysis has become less utilized, although it remains important to recognize the ability of microarray analysis as a resource to validate RNA-seq results and vice versa, and it may also be used as a basis for hypothesis testing and generation. It is important to note that data mining may subject to technological and biological biases as well as systematic errors that can impact downstream analyses [179]. A good strategy would be to combine the data mining of both microarray and RNA-seq datasets to increase the stringency and the accuracy of the DEGs identified.

The future of omics analysis lies at the interface of multi-omics integration, where genomics, transcriptomics, proteomics, metabolomics, lipidomics, as well as spatial omics can be utilized simultaneously [180]. One of the main challenges of integrative approaches concerns increased dimensionality due to the increased complexity of the omics datasets associated with the biological systems. An integrative analysis, such as independent biological integration or unsupervised machine learning, will enable the reconstruction of biological systems, with a holistic understanding of gene and protein regulation at different omic levels for translational applications [180,181]. Multi-omics data integration would provide a more sophisticated and accurate analysis for early disease detection (e.g., lysosomal dysfunction [182,183]), as well as increase precision phenotyping and personalized medicine [184,185,186,187]. It is also important to take into account pharmacogenomics to understand that individuals will respond differently to different medicines based on many biological and environmental factors. Exploring different omic datasets through established pipelines of multi-omics integration will unlock a broad range of opportunities for translational applications, including elucidation of disease mechanisms, biomarker discovery, and therapeutic development.

## Figures and Tables

**Table 1:** Summary of upregulated and downregulated genes identified from a microarray analysis of mRNA obtained from brain tissues and blood samples in AD and PD, as well as from CSF in MS. Arrows represent the direction of changes of the gene levels.

AD	Brain tissue	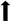 *HDAC1*, *WWTR1*, *ITGB1*, *PDGFRB*, *PLOD1*, *MAP4K4*, *NFKBIA*, *TYROBP*, *GSN*, *TIMP1*
		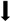 *SIRT3*, *RAB7A*, *BDNF*, *VLDLR*, *APLP2*
	Blood	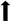 *VCAM1*, *TYK2*, *TCIRG1*, *PPP3CB*, *SNCB*, *SACS*, *GSN*, *TIMP1*
		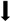 *CTSD*, *RPL11*, *SNCA*, *FKBP1B*, *BDNF*, *VLDLR*, *APLP2*
PD	Brain tissue	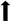 *SRRM2*
		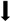 *MAPK8*, *CDC42*, *NDUFS1*, *COX4I1*, *SDHC*
	Blood	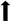 *LILRB3*, *CSF3R*, *SRRM2*
		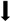 *ICAM1*
MS	CSF	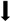 *NLRP3*, *LILRB2*, *C1QB*, *CD86*, *C1QA*, *CSF1R*, *IL1B*, *TLR2*

**Table 2:** Summary of data mining tools and programs that can be used analyze microarray, RNA-seq, and sc/snRNA-seq datasets.

Data Mining Tools/Programs	Datasets Analyzed	References
GEO2R	Microarray data	[159]
BART	Microarray data	[160]
BEAVR	RNA-seq data	[161]
RNAlysis	RNA-seq data	[162]
RNAdetector	RNA-seq data	[163]
OneStopRNAseq	RNA-seq data	[164]
IDEAMEX	RNA-seq data	[165]
ScAmpi	ScRNA-seq data	[168]
ASAP	Sc/snRNA-seq data	[169]
CReSCENT	Sc/snRNA-seq data	[170]

## Data Availability

Not applicable.
